# Acceptance of mHealth among health professionals: a case study on anesthesia practitioners

**DOI:** 10.1186/s12871-020-00958-3

**Published:** 2020-03-03

**Authors:** Hugo Carvalho, Michael Verdonck, Patrice Forget, Jan Poelaert

**Affiliations:** 1 Vrije Universiteit Brussel (VUB), Universitair Ziekenhuis Brussel (UZ Brussel), Brussels, Belgium; 2 Business Informatics Research Group, Universiteit Gent, Ghent, Belgium; 3 Institute of Applied Health Sciences, NHS Grampian, University of Aberdeen, Aberdeen, UK

**Keywords:** Anesthesia, mHealth, Smartphone application, Smartphone peripherals, Apps

## Abstract

**Background:**

mHealth, the practice of medicine aided by mobile devices is a growing market. Although the offer on Anesthesia applications (Apps) is quite prolific, representative formal assessments on the views of anesthesia practitioners on its use and potential place in daily practice is lacking. This survey aimed thus to cross-assess the Belgian anesthesia population on the use of smartphone Apps and peripherals.

**Methods:**

The survey was exclusively distributed as an online anonymous questionnaire. Sharing took place via hyperlink forwarding by the Belgian Society for Anesthesia and Reanimation (BSAR) and by the Belgian Association for Regional Anesthesia (BARA) to all registered members. The first answer took place on 5 September 2018, the last on 22 January 2019.

**Results:**

Three hundred forty-nine answers were obtained (26.9% corresponding to trainees, 73.1% to specialists). Anesthesiologists were positively confident that Apps and peripherals could help improve anesthesia care (57.0 and 47.9%, respectively, scored 4 or 5, in a scale from 0 to 5). Trainees were significantly more confident than specialists on both mobile Apps (71.2% and 51.8%, respectively; *p* = 0.001) and peripherals (77.7% and 45.1%, respectively; *p* = 0.09).

The usefulness of Apps and Peripherals was rated 1 or below (on a 0 to 5 scale), respectively, by 9.5 and 14.6% of the total surveyed population, being specialists proportionally less confident in Smartphone peripherals than trainees (*p* = 0.008). Mobile apps are actively used by a significantly higher proportional number of trainees (67.0% vs. 37.3%, respectively; *p* = 0.000001).

The preferred category of mobile Apps was dose-calculating applications (39.15%), followed by digital books (21. 1%) and Apps for active perioperative monitoring (20.0%).

**Conclusions:**

Belgian Anesthesia practitioners show a global positive attitude towards smartphone Apps and Peripherals, with trainees trending to be more confident than specialists.

**Trial registration:**

ClinicalTrials.gov database Identifier: NCT03750084. Retrospectively registered on 21 November 2018.

## Background

Smartphones are a ubiquitous phenomenon. The massive production of these multisensory devices has reduced their overall cost and increased their societal penetrance. Their high processing capacity entails a rather useful leverage for healthcare in general, a sector where data is abundant and its processing relevant for clinical decision-making [1, 2]. These advantageous features have been quickly assimilated by anesthesiologists, and dedicated anesthesia applications for various perioperative purposes have been continuously sprouting [3]. Medical device manufacturers have been similarly leveraging on this versatility in order to commercialize smartphone plug-in devices (also known as smartphone peripherals) that can be used for diagnostic purposes. These include, among others, echography probes (Butterfly™, Clarius™, Philips Lumify™), video laryngoscopes (Airtraq™ Phone adapter) and stetoscopes (StethIO™).

Commonly referred to as “mHealth” (abbreviation for Mobile Health), the practice of medicine aided by mobile devices is a growing market. In the United States of America (USA), this sector has been estimated to be worth more than 28 billion dollars in 2018, and predicted to surpass the 100 billion dollar barrier by 2023 [4]. Despite its exponential growth, regulation has been lagging behind and Food and Drug Administration (FDA) data shows that from a pool of more than 150,000 mobile applications (Apps) within the Health/Wellness category, only around 200 (0.1%) had been submitted to standardized governmental validation procedures [5].

Despite the high mobile applications output, formal surveying of the views of anesthesia providers on these applications is scarce [3, 5]. Green et al. have conducted one of the most complete, although non-representative, studies on the pattern of utilization of smartphone applications by anesthesiologists in the USA [3].

The aim of the present survey was to specifically cross-assess the Belgian anesthesia population on this same subject, as well as to discuss the results with respect to the current legal European framework around mHealth.

## Methods

The present study was approved by Ethical Committee of the Universitair Ziekenhuis Brussel, Belgium (Reference 2018/435, B.U.N. 143201837927), and registered at the ClinicalTrials.gov database (Identifier: NCT03750084). The survey was specifically developed for the present study and has not been published elsewhere. The targeted population referred to active (practising) Belgian anesthesiologists (both trainees and specialists), and the a priori established aim was the assessment of the confidence level of this population on both smartphone applications and dedicated smartphone peripherals within daily anesthesia practice. Future development expectations/desires were also to be assessed. Assessment of user experience was not within the scope of the present study.

The survey was not piloted and was exclusively distributed as an online anonymous questionnaire (Google™ Forms platform) for traceability purposes. Sharing took place via hyperlink distribution by the Belgian Society for Anaesthesia and Reanimation (BSAR) and by the Belgian Association for Regional Anaesthesia (BARA) to all registered members. The first answer took place on 5 September 2018, the last on 22 January 2019.

The original survey is available as a supplementary file as well as online at: https://goo.gl/forms/7job24qgFOPXpUD12

It was divided in two main sections: one pertaining to Smartphone Applications themselves, another to Smartphone Peripherals. Each section was identically subdivided and sequentially evaluated the following topics:

- Confidence that Smartphone applications / peripherals can help improve Anesthesia care and why.
- Phase of perioperative care in which Smartphone applications / peripherals are most useful.
- Which sort of Smartphone applications appeal the user the most.
- Which Smartphone applications / peripherals the user employs in his/her daily practice.
- What are the user’s wishes on the development of future Smartphone applications / peripherals.

The survey has been structured based on the Technology Acceptance Model (TAM), an information systems theory that describes how users come to accept and use new technologies [6, 7]. The model suggests that when users are presented with a new technology, two primary factors influence their decision about how and when they will use it: (1) Perceived ease of use, which is determined by the degree to which a person believes that using a particular technique would be free of effort; and (2) Perceived usefulness, referring to the degree in which a person believes that a technique will be effective in achieving the intended modelling objective. The aforementioned model and associated measures were concordantly translated into the current survey to assess how respondents perceived the acceptance of mobile applications and peripherals within anesthesia. More specifically, participants had to answer several questions – using multiple-item scales with a Likert structure – which measured both the perceived usefulness and perceived ease of use. The reliability and validity of these type assessments has been assessed in several similar research efforts [8–11].

Questions were in their majority presented to the surveyees with a categorical structure. Dichotomous, nominal and contingency questions were used to categorize individuals as well as the contextual use of Apps and Peripherals. Confidence levels were assessed by a Likert-type scale with balanced keying in order to allow for discrete quantitative comparisons. A score of 3 was considered the positivism transition point (considered to “Improve Anesthesia Care”), and a score of 4 or 5 was considered as positively trending confidence. Optional open text questions were used for detailing the reasons for the selected subjective confidence level.

Data reporting for the total population and for each subgroup (consultants/trainees) was descriptive in nature and precision reported with 95% Confidence Intervals [95%CI]. The inter-group confidence level comparisons based on the multi-point (ordinal) rating scales levels were carried out by means of binary reconversion of the Likert scale into two mutually exclusive intervals (one encompassing the ratings 0 to 3, and the second 4 to 5), and by sequential non-parametric analysis by means of Chi-square testing with a significance cut-off of 0.05. Identical methodology was used for the analysis of inter-group differences in terms of active use of Apps or Peripherals to aid Anesthesia care.

## Results

**Fig. 1:**
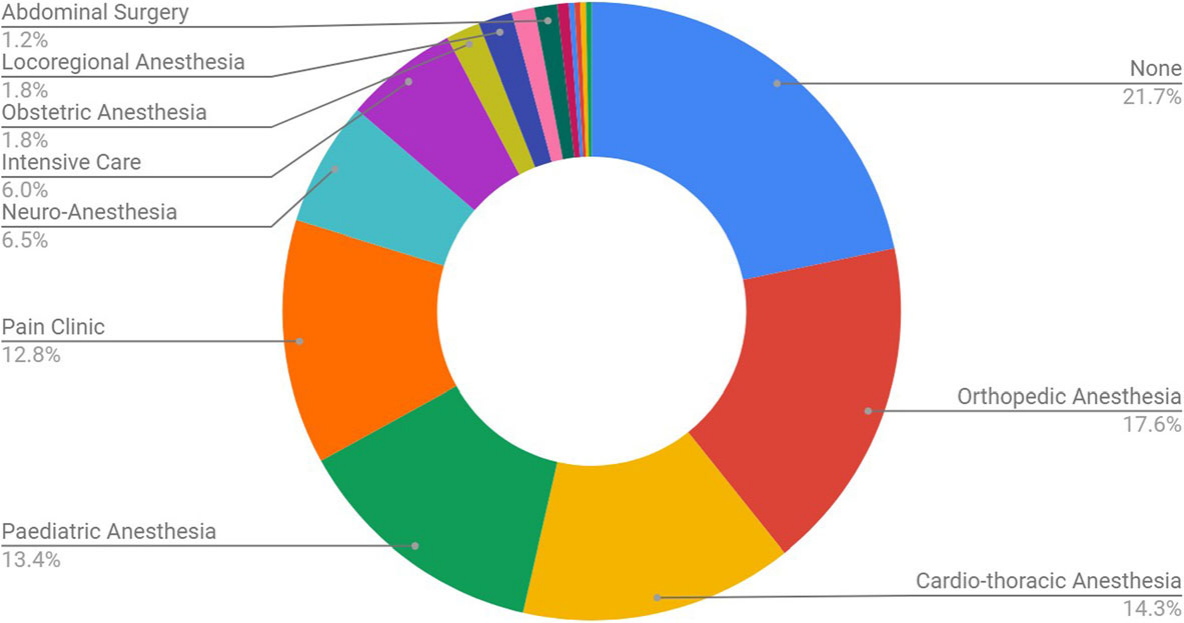
Subspecialty stacked distribution of responding Anesthesia Specialists (one specialist can be accounted for more than once if he holds multiple subspecialty competences). Percentages represent the total number of surveyees per specific category relative to total number of surveyees

A total of 349 answers were obtained. Ninety-four (26.9%) responses were of Belgian Anesthesia trainees, 255 (73.1%) from Belgian Anesthesia consultants. A majority of the answering specialists (21.7%) had no dedicated subspecialty activities or were all-round specialists (Fig. 1). Anesthesiologists with an orthopedic anesthesia subspecialty accounted for 17.6% of the total, followed by cardio-thoracic anesthesiologists (14.3%), Pediatric Anesthesiologists (13.4%), Pain Clinic specialists (12.8%), Neuro-anesthesiologists (6.5%) and Intensive care specialists (6.0%). The remainder subspecialties were underrepresented (less than 1.8%).

**Fig. 2:**
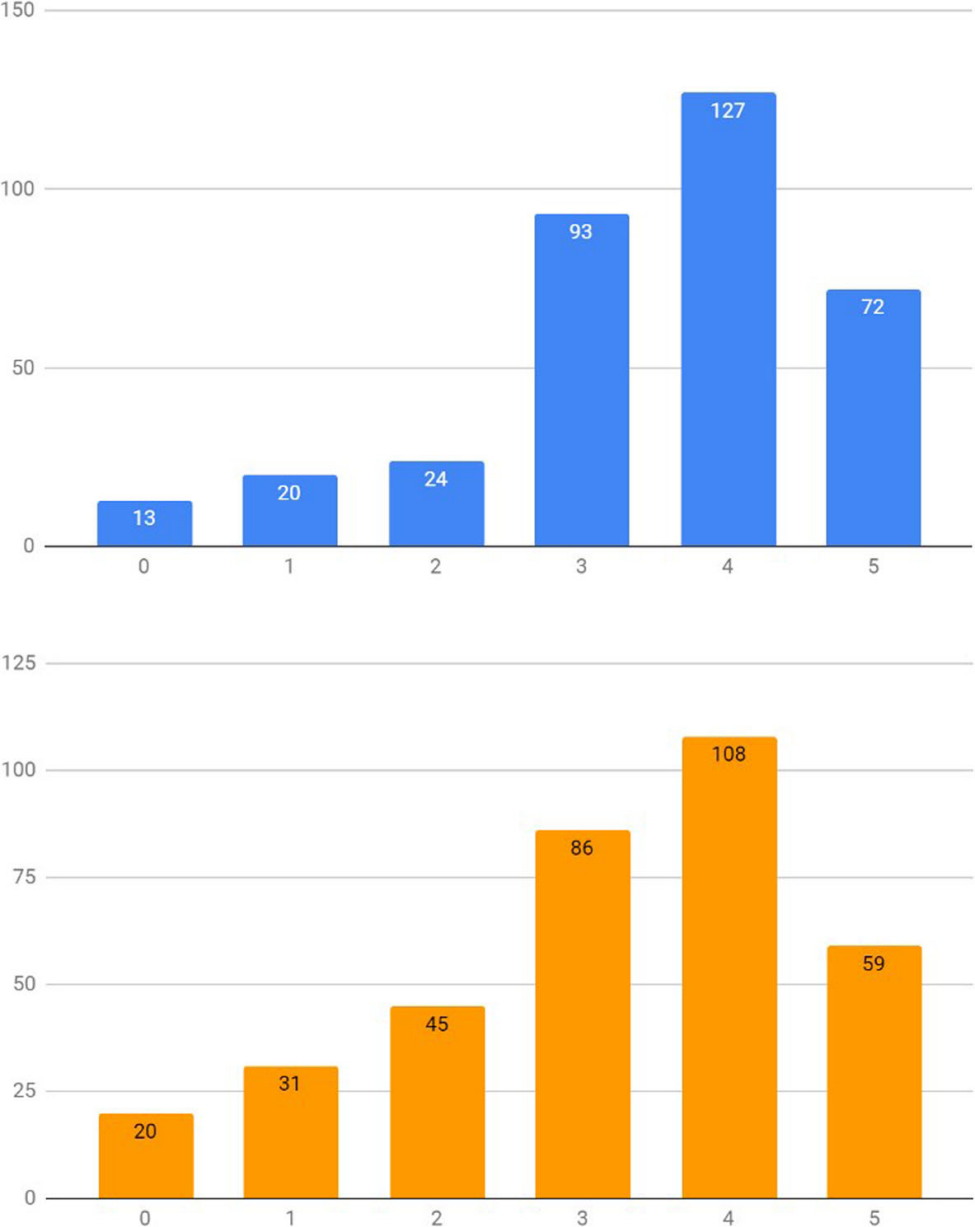
Apps (left - blue) vs Peripherals (right - orange) - Confidence level (scale: 0 to 5). x axis – Confidence level category, y axis – absolute number of survey answers (“How confident are you that Smartphone Apps can help improve anesthesia care?” / “How confident are you that combining your smartphone with a dedicated monitoring peripheral can help improve anesthesia care?”)

**Fig. 3:**
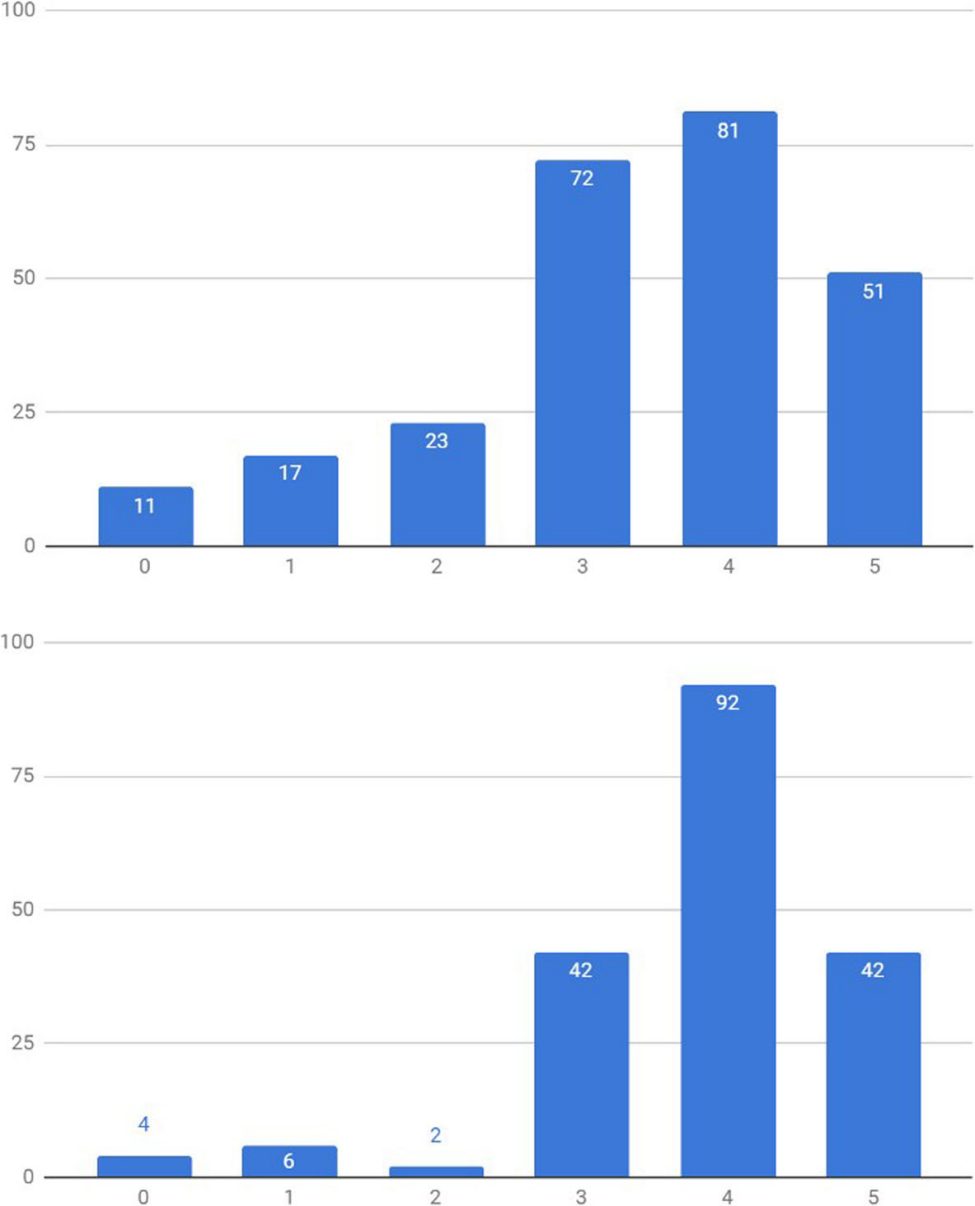
Apps Confidence level (scale: 0 to 5): Specialists (left) vs Trainees (right). x axis – Confidence level category, y axis – absolute number of survey answers

**Fig. 4:**
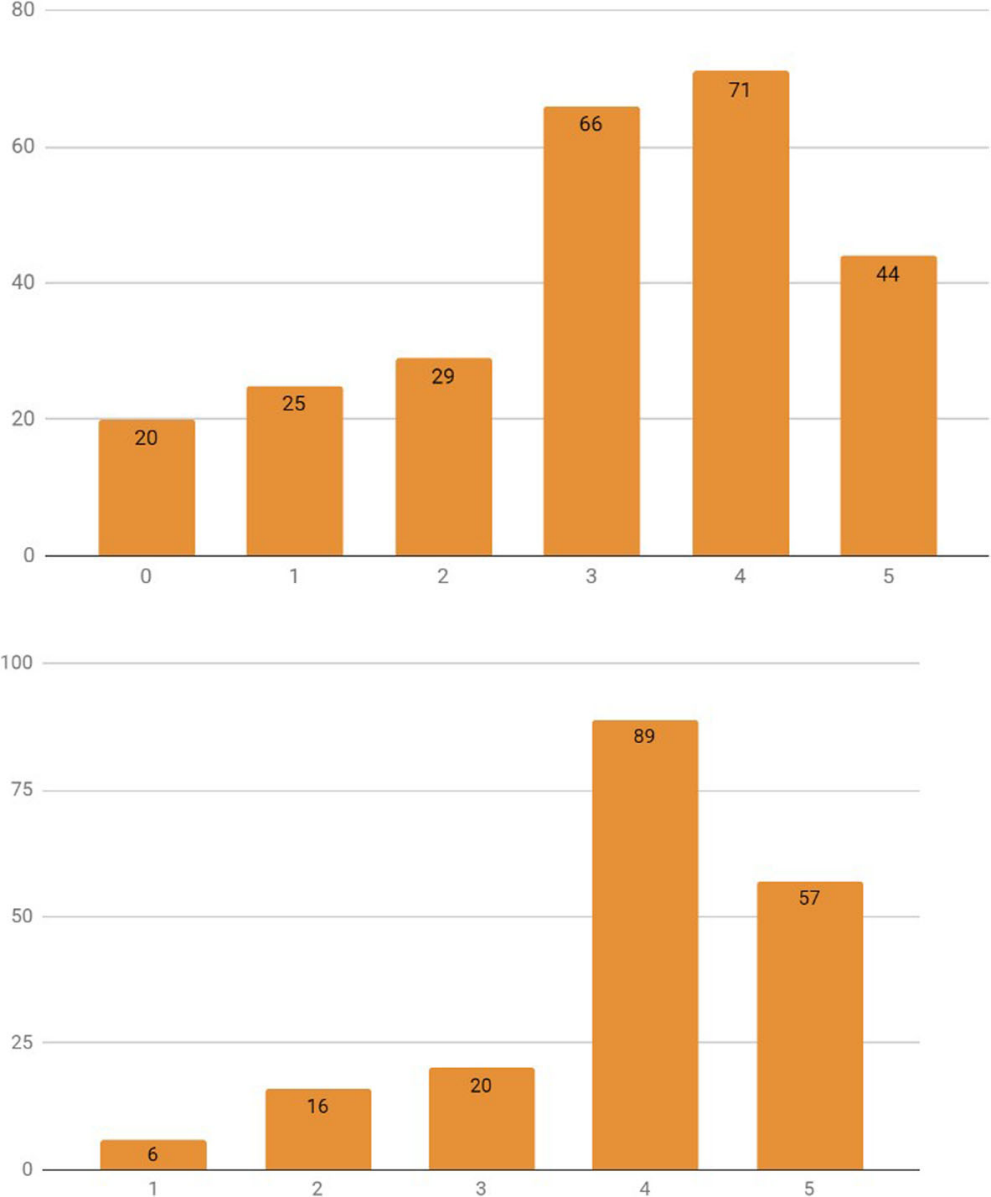
Peripherals Confidence level (scale: 0 to 5): Specialists (left) vs. trainees (right). x axis – Confidence level category, y axis – absolute number of survey answers

When asked on how confident they were that Smartphone Applications (Apps) or Smartphone Peripherals (Peripherals) could improve anesthesia care, a majority of the Belgian anesthesiologists were positively confident (score of 4 or 5, on a 0 to 5 categorical scale) that these could indeed help improve anesthesia care (57.0% [95%CI: 51.8–62.2%] and 47.9% [95%CI: 42.7–53.1%], respectively, scored 4 or 5) (Fig. 2). When subanalyzing the data per experience group, anesthesia trainees demonstrated a significantly higher degree of optimism (score of 4 or 5, out of 5) on Mobile Apps compared to consultants (71.3% [95%CI: 62.1–80.4%] and 51.8% [95%CI: 45.6 57.9%], respectively) (X^2^ [1, *N* = 349] = 10.6696, *p* = 0.001) (Fig. 3). This positivity trend was maintained for Smartphone peripherals (77.7% [95%CI: 69.3–86.1%] and 45.1% [95%CI: 39.0–51.2%], respectively), although no statistical significance was retained (X^2^ [1, *N* = 349] = 2.8754, *p* = 0.090) (Fig. 4).

Nine and a half percent [95%CI: 6.4–12.6%] of the surveyees (consultants and trainees combined) rated Apps’ usefulness in Anesthesia as 1 or below (on a 0 to 5 scale), and 14.6% [95%CI: 10.9–18.3%] gave the same rating when asked about Peripherals. Inter-group analysis for this rating showed no statistical significance between trainees and consultants for Apps (X^2^ [1, *N* = 349] = 2.5711, *p* = 0.108833). On the other hand, smartphone peripherals were significantly more negatively rated by consultants than by trainees (X^2^ [1, N = 349] = 6.9839, *p* = 0.008225).

From all the responders, 45. 3% [95%CI: 40.0–50.5%] actively used Apps to aid their anesthesia practice, compared to only 3.2% [95%CI: 1.3–5.0%] that use Peripherals in their daily anesthesia practice. Again, subanalysis of the answers per training group showed that trainees actively use mobile apps in a significantly higher proportion when compared to consultants (67.0% [95%CI: 57.5–76.5%] and 37.3% [95%CI: 31.3–43.2%], respectively) (X^2^ [1, N = 349] = 24.5615, *p* = 0.000001) (Table 1). No statistically significant inter-group difference was found in terms of active peripherals use) (X^2^ [1, N = 349] = 0.4421, *p* = 0.506108) (Table 1).

**Table 1:** Active Usage of Mobile Apps and Peripherals per subgroup

	Apps	Peripherals
Specialists	95 (37.3%)	9 (3.5%)
Trainees	63 (67.0%)	2 (1.2%)
Total	158 (45.3%)	11 (3.2%)

Cell values represent the absolute number of individuals. Within parenthesis the percentages are relative to total of individuals within the same cell line-group (i.e., relative to either specialists, trainees or total surveyees)

**Fig. 5:**
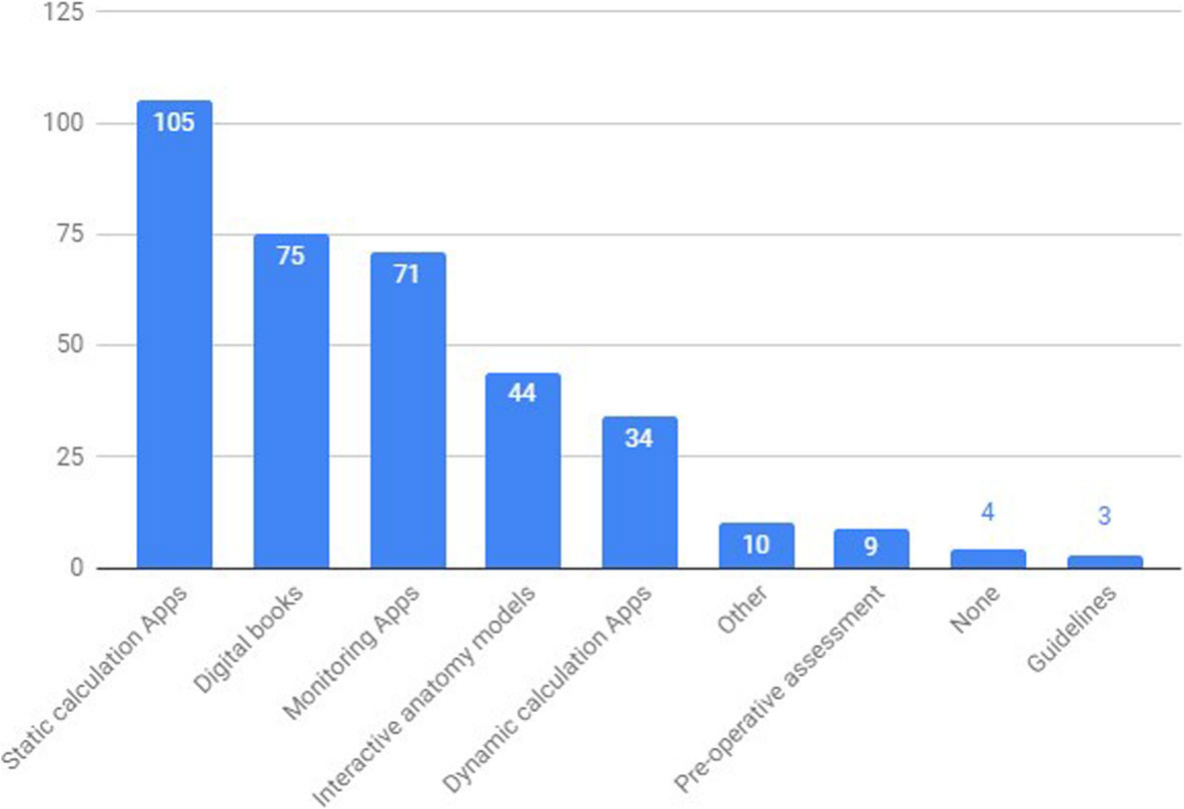
Categorization of the most appealing Apps (“Which kind of Apps appeal you the most?”). x axis – App category, y axis – absolute number of survey answers

When questioned on which App category was more appealing, 39.15% [95%CI: 34.0–44.3] of total responders gave preference to dose-calculating applications (dynamic [TCI modelling] and static [fixed dose calculation] apps). The next bigger App preference were Digital Books (21.12% [95%CI: 16.8–25.4%]), followed by Applications used for perioperative monitoring (20.0% [95%CI: 15.8–24.2%]) and interactive anatomy models (12.39% [95%CI: 8.9–15.8%]) (Fig. 5).

**Fig. 6:**
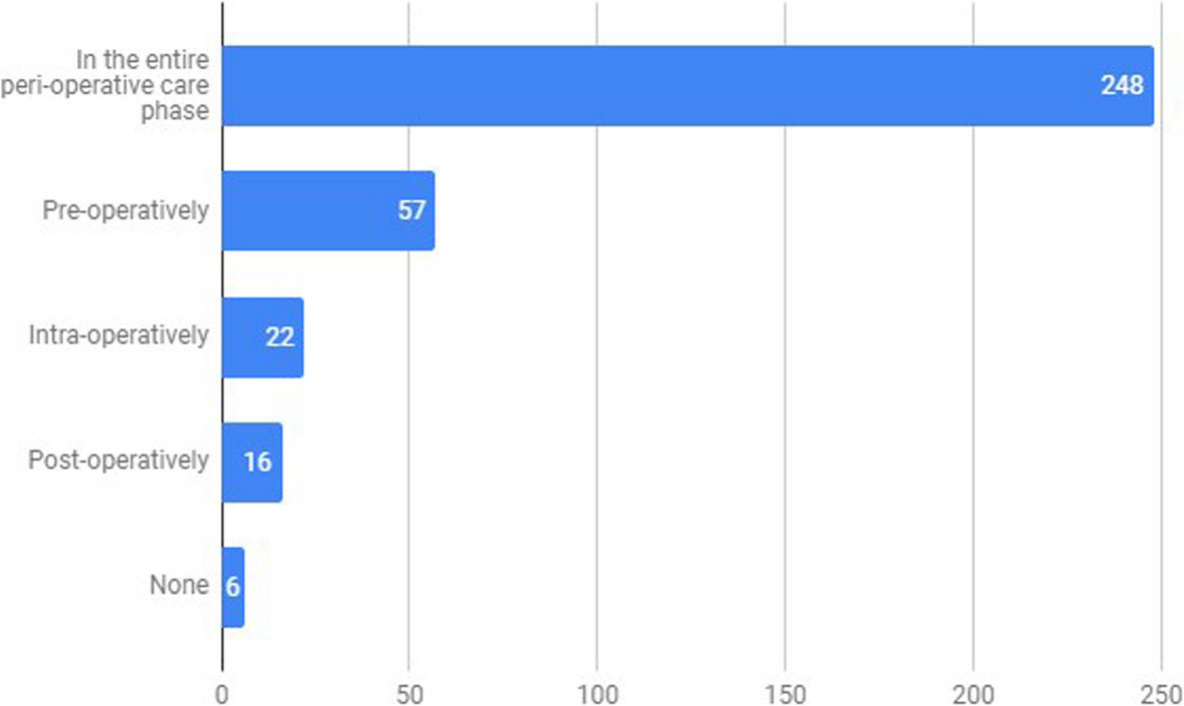
Phase in which Smartphone Apps can be more useful (“In which phase of perioperative care can Smartphone Apps be more useful?”). x axis – absolute number of survey answers, y axis – Perioperative phase category

**Fig. 7:**
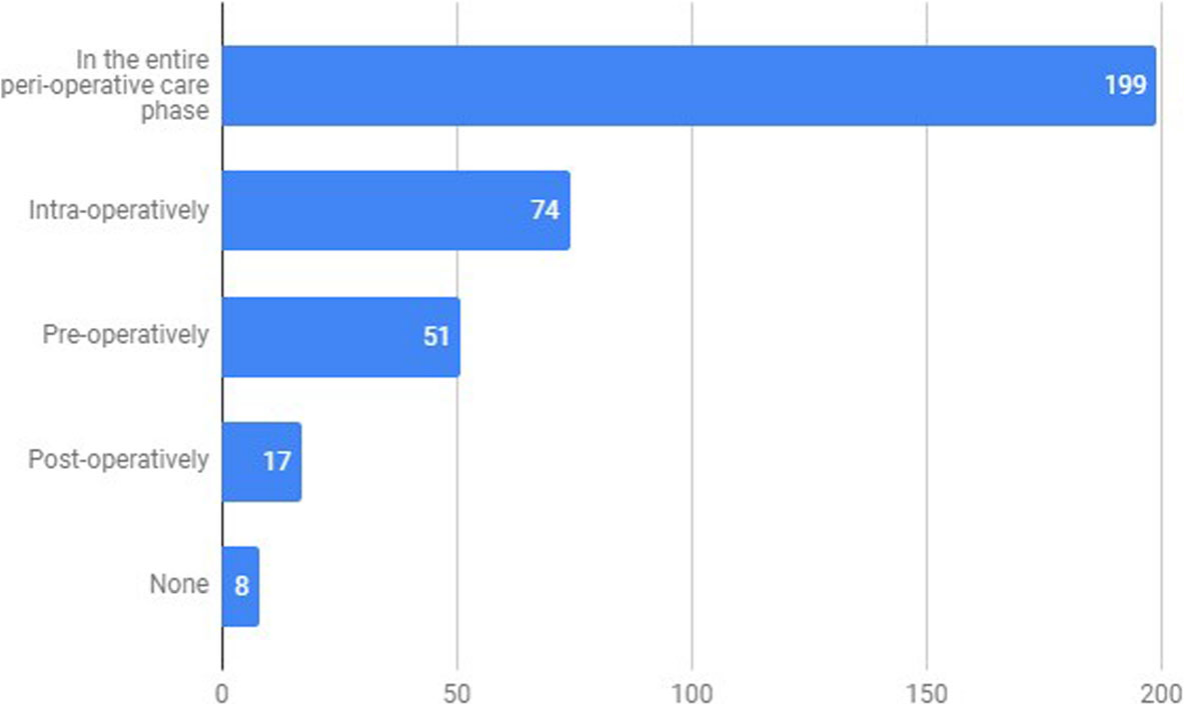
Phase in which Smartphone Peripherals can be more useful (“In which phase of perioperative care can Smartphone Peripherals be more useful?”). x axis – absolute number of survey answers, y axis – Perioperative phase category

Concerning the perioperative care phase in which Applications or Peripherals could be more useful, 71.1% [95%CI: 66.3–75.9%] and 57.0% [95%CI: 51.8–62.2%], respectively, considered them to have a potential use in all phases of the perioperative care (Figs. 6 and 7).

**Fig. 8:**
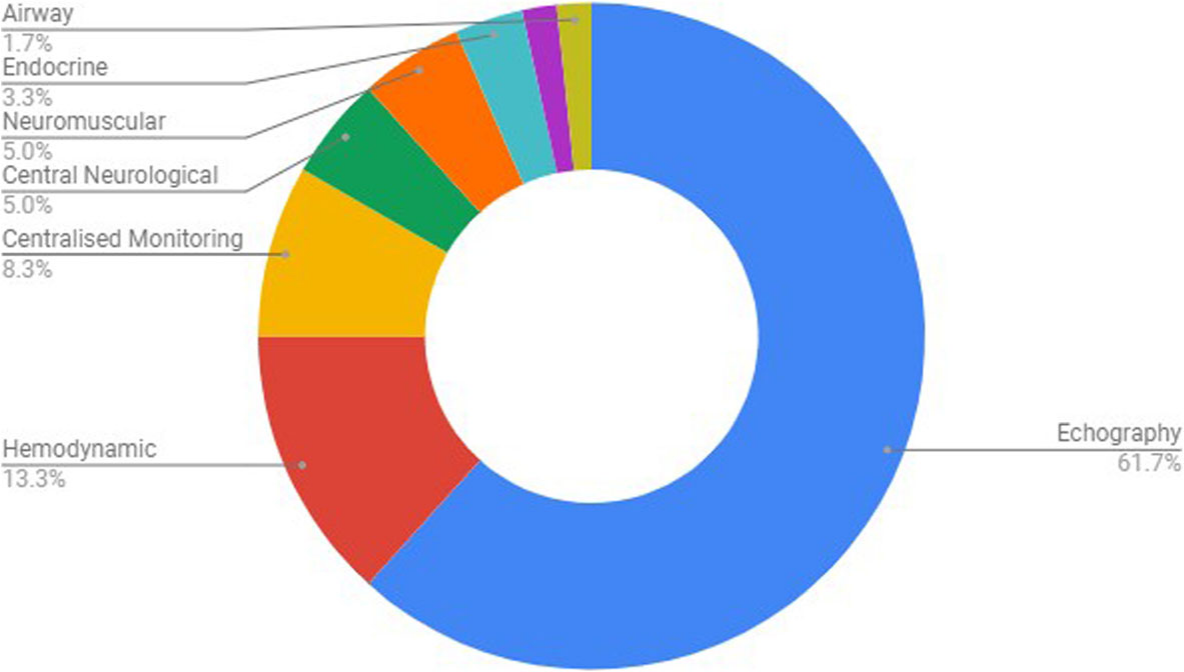
Wishes for smartphone peripheral device development per monitoring category (“Which peripherals would you like to see developed in the coming future?”). Percentages represent the total number of votes per specific category relative to total number of votes

The categories in which anesthesiologists would like to see development of smartphone peripheral devices are illustrated in Fig. 8.

## Discussion

In general, these survey results agree with the findings of Green et al. on the American anesthesiologists population, where apps enjoy a significant degree of confidence and are believed to have a potential use on all phases of perioperative care [3]. Peripherals also enjoy a high confidence on potential use, rating 47.9% [95%CI: 42.7–53. 1%] of the responders their confidence as 80% or higher that these can be useful in Anesthesia care. Nine and a half percent [95%CI: 6.4–12.6%] of the surveyees rated Apps’ usefulness in anesthesia as 1 or bellow (on a 0 to 5 scale), and 14.6% [95%CI: 10.9–18.3%] gave the same rating when asked about Peripherals. Thus, Apps enjoy both a greater degree of optimisms as well as a lower degree of disbelief in comparison to Peripherals. The reasons for this discrepancy were not evaluated by this questionnaire, but one can speculate that the underdeveloped regulated market of smartphone peripherals for diagnostic aid is still not firmly established within today’s anesthesia practice. Although the major players have already created a dedicated peripherals market branch (f.e., the Philips Lumifym® portable echography series), convincing of practitioners on their usefulness is still needed. Curiously, when asked on which peripherals they wanted to see developed, 61.7% of the anesthesiologists answered “Echography”. This is nonetheless one of the more exploited areas in terms of Anesthesia smartphone peripherals, and has been explored both by the major players in the medical device industry (Philips™, Airtraq™), as well as by less known and upcoming competitors (Clarius™, Butterfly™). From the total of 66 individuals providing a written rationale for their confidence levels on mobile peripheral devices, 6 (9.1%) suggested that although they did know of the existence of such products, they still found them economically inaccessible. Other, however, suggested they had no knowledge of such devices. Another possible reason that might contribute to the greater disbelief possibly relates to the medical use of an originally partially non-medical device. Although it seems logical that controlled CE-labelling (Conformité Européenne) of smartphone peripherals for medical use might help overcome this phenomenon, a subjective factor cannot be underestimated. Just like heavy, well designed and good fitting over-head headphones feel subconsciously better than in-ear equivalents, traditional anesthesia monitors might still convey more confidence [12].

Another curious pattern observed on the surveyees’ answers was the fact that although 57.0% [95%CI: 51.8–62. 2%] considered Apps useful (classification of 4 or 5 out of 5), only 45.3% [95%CI: 40.0–50.5%] reported actually using them in their daily practice. The gap was proportionally bigger when analysing smartphone peripherals (47.9% [95%CI: 42.7–53.1%], and 3.2% [95%CI: 1. 3–5.0%], respectively), although the latter easier to justify in light of the underdeveloped smartphone peripherals market. This Smartphone “Confidence - Active use” gap might be explained by a yet unripe and ununiformed anesthesia app market. A perceptive phenomenon of unrealistic and consequently unfulfilled expectations by users must also be considered as possible, although formal prospective user experience assessments are needed for this purpose.

In line with the study of Green et al., dosage apps were chosen by the majority as the most useful [3]. Digital books and perioperative apps followed. Such choice pattern is not counter-intuitive considering the still limited interactivity between handheld devices and anesthesia monitoring devices, although such justification is purely speculative as formal assessments to this point are lacking. The increasing focus on portability and cross-connectivity might lead to a pattern change, and future studies would be useful to analyse a trend shift.

The observed positive disposition towards mHealth usage as well as its focus on mobile apps is apparent on indexed literature analysis. In fact, notwithstanding a possible positive publication bias, the publication of mHealth applications within all domains of healthcare has been steadily increasing [13]. Curiously, and notwithstanding the fact that representative reports on global mHealth usage patterns are lacking, analysis of an individual application’s trends have shown a higher penetration of these low cost aids in low income countries [14].

Within the anesthesia domain, developed applications range from crisis management support apps, to post-operative pain assessment, but also to non-medical topics such as logistic optimisation of Operation room supplies [15–17]. Most of these reports are descriptive and lack usability testing to allow a direct comparison to the present study’s results.

As opposed to the study of Green et al., our group found significant differences between anesthesia trainees and specialists. Although there was a global positivity trend towards mobile apps in both groups, training anesthesiologists displayed a significantly higher confidence on mobile apps than consultants (71.3% vs 51.8%, respectively, *p* = 0.001). This positivity trend was similarly true for smartphone peripherals (45.1% vs 77.7%, respectively), although this difference didn’t retain statistical significant on further difference testing (*p* = 0.09). Besides the evident cultural and contextual medical practice differences between the sampled subjects (American vs. European), the collected data on both studies is insufficient to put forward a phenomenological explanation.

According to data from the Belgian National Institute for Health and Disability Insurance (RIZIV / INAMI), in the beginning of 2016, Belgium had 2441 active anesthesia specialists (certified specialists and trainees) [18]. This sets this survey’s cross-sectional percentage at 14.2% of the total active Belgian anesthesiologists, 13.2% of the certified Belgian anesthesiologists, and 17.5% of the Belgian anesthesiology trainees. Concerning the accredited specialists (diploma-holding), it is however not known if all of them are dedicated in exclusivity to anesthesia-related fields such as Intensive care, Emergency or Chronic Pain. It is thus possible that the representability percentage of this survey is different than calculated, although practically very difficult to confirm.

These definitely promising technologies are increasingly being introduced in our daily practice and play an important facilitating role. However, one must not forget that these freely available tools are not always subject to formal approval procedures that scientifically validate their clinical use. Most of these are part of the off-label/“use at own risk” category (commonly referred to as “Grey Area Apps”) - applications freely available without formal evaluation of their function for their stated (medical) use [8]. Taking this into mind, the European Union (EU) has created between 2016 and 2017 a workgroup for the development of mHealth assessment guidelines [19]. However, the group was not able to endorse concrete guidelines by failure to reach a minimal intra-group consensus [20]. As of this moment, Grey Area Apps remain unregulated. There is, however, a non-binding “privacy code of conduct on mobile health apps” that outlines the core values that should guide mobile health application development [21]. It provides a theoretical competitive advantage against non-conform Applications and speeds up an eventual CE-label request. As for applications aiming for a formal regulated national market entry, compliance with the EU regulation 2017/745 (from 5 April 2017) is mandatory. Together with the EU norm 2017/746, they regulate the European market of medical devices since May 2017. European Union state members fall, thus, under these norms.

It is self-evident that mobile Applications and Peripherals are quickly permeating all phases of Healthcare, with the right steps are being taken for their scientific, national and intracontinental integration [22–33]. Peripherals still lag behind mobile applications although they constitute an economically and clinically important area. Care must still be taken considering the majority of available Apps fall within the unregulated category of “Grey Area Apps”. Last, but not least, care is necessary in avoiding over-reliability/dependency on Apps, with the consequent side-tracking of basic clinical skills. Notwithstanding this warning note, the education potential of apps as supplement to classical learning techniques is increasingly being explored with some educational centers incorporating such solutions within anesthesia training programmes [34]. The development of applicationS should ideally use a user-centered design for and optimal and successful adoption [15].

The present study is limited in the fact that it doesn’t directly address user experience. Initially designed primarily to address the acceptance of Apps and Peripherals, user experience and expectations were left out in the need for a compromise between brevity and completeness. A mixed-method experience analysis would be a relevant top-up survey that would allow this quantification as well as to potentially guide App and Peripheral development based on end user experience and expectations. Secondly, the survey is further limited in the fact that the transversal population assessment was estimated at 14.3% (349 out of 2441 active anesthesia specialists), raising the obvious concern of non-responder bias. Finally, the fact that digital books have been considered Apps might constitute a classification bias depending on surveyee interpretation. In fact, digital books might also come in non-app form (for example as *.pdf or *.chm format), which could potentially affect the perception of the respondents.

## Conclusions

Belgian Anesthesia practitioners show a positive attitude towards smartphone-based solutions within Anesthesia care, mirroring international reported trends within other medical sectors. There is evidence of an international recognition of the potential of these technologies within the healthcare domain, with consequently rising regulatory efforts from medical societies and national legislative bodies.

## Supplementary information

**Additional file 1.** Original survey used for the present study.

## Data Availability

The datasets used and/or analysed during the current study are available from the corresponding author on reasonable request.

## Abbreviations

Apps: Mobile Applications
BARA: Belgian Association for Regional Anesthesia
BSAR: Belgian Society for Anesthesia and Reanimation
CE: Conformité Européenne
EU: European Union
FDA: Food and Drug Administration
mHealth: Mobile Health
RIZIV/INAMI: Belgian National Institute for Health and Disability Insurance
TAM: Technology Acceptance Model
USA: United States of America
VUB: Vrije Universiteit Brussel
