# Supplementary material for: Acceptance of mHealth among health professionals: a case study on anesthesia practitioners
Source: BMC Anesthesiol. 2020 Mar 3;20:55. doi: 10.1186/s12871-020-00958-3 (PMC7053134; doi:10.1186/s12871-020-00958-3)
Supplement: Supplementary file 1 — Additional file 1. Original survey used for the present study. [file 12871_2020_958_MOESM1_ESM.pdf]

Anesthesia and m-Health

*Required

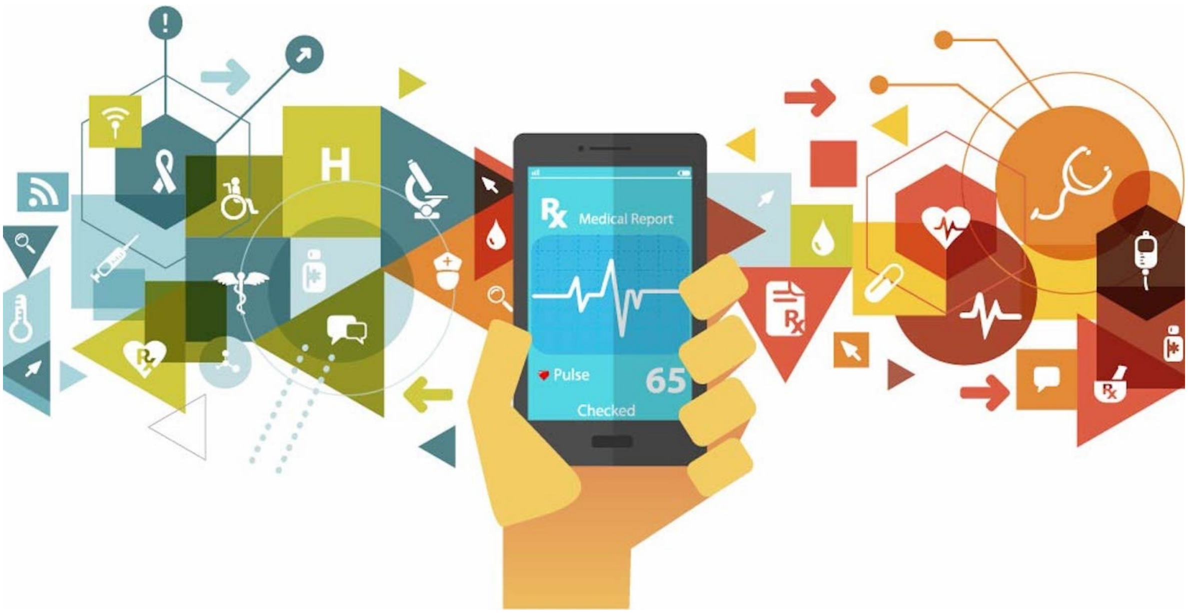

Aim

This 3 minute survey aims to better define the wishes/needs of the Anesthesiologist within the field of mobile technology.
What would help you improve your daily practice ?

Specialty Degree *

- Trainee / Specialty Registrar
- Recognized Specialist / Consultant

Country of Practice *

Your answer

Subspecialty *

- [ ] Paediatric Anesthesia
- [ ] Cardio-thoracic Anesthesia
- [ ] Neuro-Anesthesia
- [ ] Orthopedic Anesthesia
- [ ] Pain Clinic
- [ ] None
- [ ] Other:

Smartphone Applications (Apps)

How confident are you that Smartphone Apps can help improve anesthesia care ? *

	0	1	2	3	4	5	
Apps will NOT improve anesthesia care	○	○	○	○	○	○	Apps will improve anesthesia care

Why ? (optional)

Your answer

In which phase of peri-operative care can Smartphone Apps be more useful ? *

- ◯ Pre-operatively
- ◯ Intra-operatively
- ◯ Post-operatively
- ◯ In the entire peri-operative care phase
- ◯ Other:

Which kind of Apps appeal you the most ? *

- ◯ Digital books
- ◯ Interactive anatomy models
- ◯ Static calculation Apps (fixed dose calculation)
- ◯ Dynamic calculation Apps (TCI modelling)
- ◯ Monitoring Apps (sensor use for monitoring purposes)
- ◯ Other:

Do you use any mobile Apps to aid your Anesthesia Care ? *

- ◯ Yes
- ◯ No

If yes, which ?

Your answer

Smartphone Peripherals

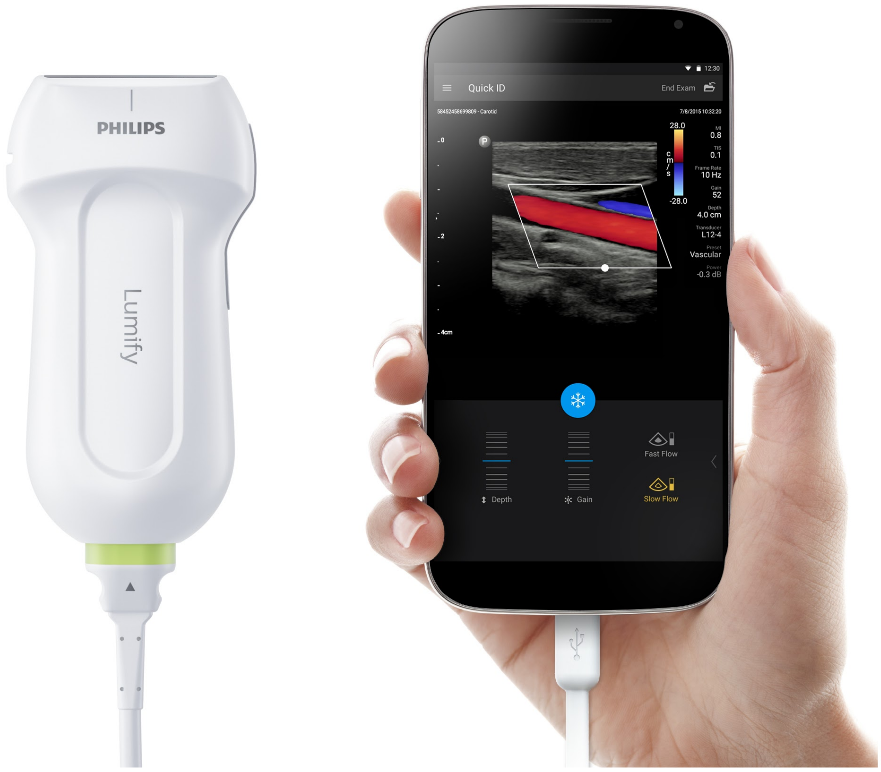

How confident are you that combining your smartphone with a dedicated monitoring peripheral can help improve anesthesia care ? *

	0	1	2	3	4	5	
Smartphone Peripherals will NOT improve anesthesia care	○	○	○	○	○	○	Smartphone Peripherals will improve anesthesia care

Why ? (optional)

Your answer

In which phase of peri-operative care can Smartphone Peripherals be more useful ? *

- Pre-operatively
- Intra-operatively
- Post-operatively
- In the entire peri-operative care phase
- Other:

Do you use any smartphone Peripherals to aid your Anesthesia Care ? *

- Yes
- No

If yes, which ?

Your answer

Your wishes and expectations

Which apps would you like to see developed in the coming future ? (optional)

Your answer

Which peripherals would you like to see developed in the coming future ? (optional)

Your answer

Consent

Do you consent that we use your anonymous answers for publication purposes ? *

- ◯ Yes
- ◯ No

* Consent might be withdrawn at anytime by contacting the author at: hnogueir@vub.ac.be

Page 1 of 1 Submit

Never submit passwords through Google Forms.

This content is neither created nor endorsed by Google. Report Abuse - Terms of Service - Privacy Policy

Google Forms
